# Editorial: Impact of COVID-19 on HIV/STI screening, prevention, and treatment

**DOI:** 10.3389/frph.2023.1228325

**Published:** 2023-07-17

**Authors:** Maria Pyra, Phetole Walter Mahasha

**Affiliations:** ^1^ Feinberg School of Medicine, Northwestern University, Chicago, IL, United States; ^2^ Grants, Innovations and Product Development (GIPD) Unit, South African Medical Research Council, Cape Town, South Africa; ^3^ Department of Nuclear Medicine, Faculty of Health Sciences, School of Medicine, University of Pretoria, Pretoria, South Africa

**Keywords:** HIV, COVID-19, Sexually tranmistted infections, testing, Pandemic (COVID-19)

**Editorial on the Research Topic** Impact of COVID-19 on HIV/STI screening, prevention, and treatment

The COVID-19 pandemic has impacted and continues to impact healthcare access in a variety of ways. Globally, the pandemic led to changes in access to HIV treatment and prevention, as well as to HIV/STI testing. This Research Topic covers 4 papers that explore the impact of COVID-19 pandemic in more detail. From Zambia, authors Kafwanka et al. examined the impact of the pandemic on ART adherence among those living with HIV, with the pandemic starting contemporaneously with a national Undetectable = Untransmittable (U = U) campaign. Among those with at least 6 months on treatment, and across a range of regimens, viral load suppression was more common among adults starting treatment during the pandemic compared to the 9 years before the pandemic. The authors provide a thoughtful discussion on the ways the pandemic interfered with adherence and emphasize the need to create a resilient healthcare system. From the Netherlands, Goense et al. investigated the use of home-testing to address gaps in sexual health services during the pandemic using a behavior change framework. Among MSM who had previously attended state-run sexual health clinics, the authors found many had continued testing during the pandemic. Normative beliefs and intentions were all positive for the use of home testing, with no variation by self-reported sexual behavior. The authors conclude with a discussion on how to integrate home testing with the clinic model. Two papers from the United States in Chicago explore changes in testing during different phases of the pandemic. Authors Stanford et al. analyzed STI testing and diagnoses at a University Emergency Department (ED), which serves a socio-economically vulnerable population. They have reported that, while testing declined during the earliest months of the pandemic and returned to expected patterns by the end of the first year of the pandemic, positivity rates for STIs increased during the same early period before returning to normal (with chlamydia rates remaining high). The authors include a discussion of how to better support the use of STI testing in ED, especially to support marginalized populations. Last, a paper by Pyra et al., examined trends in HIV and STI testing at a Chicago federally-qualified health center (FQHC) over three phases of the pandemic. Their analysis also found a decline in testing during the earliest phase of the pandemic, followed by increases to almost pre-pandemic levels in late stages of the pandemic, although some trans and nonbinary populations may still be testing at lower levels.

Taken together, these studies clearly illustrate that, across countries, the COVID-19 pandemic had a significant impact on access to HIV and STI healthcare services, predominantly during the earliest months of the local response. While some aspects—like STI testing—appear to be returning to pre-pandemic levels, the impact of untreated diseases or delayed HIV suppression will take longer and more effort to reverse. These papers suggest areas to strengthen healthcare systems against future interruptions—which populations may most benefit when access is a limited resource, how to improve access through novel testing and care approaches, and how to build a more resilient healthcare system.

